# Supplementary material for: Cell Surface Expression of Nrg1 Protein in Candida auris
Source: J Fungi (Basel). 2021 Mar 31;7(4):262. doi: 10.3390/jof7040262 (PMC8066560; doi:10.3390/jof7040262)
Supplement: Supplementary file 1 [file jof-07-00262-s001.pdf]

Cell surface expression of Nrg1 protein in *Candida auris*

Anuja Paudyal [1] and Govindsamy Vediyappan [2,*],

Division of Biology, Kansas State University, Manhattan KS 66506, USA

[1] anuja@ksu.edu; [2] gvediyap@ksu.edu *Correspondence: gvediyap@ksu.edu

Figure S1. Determination of specificity of the anti-*Cal*Nrg1 antibody. The *Cal*Nrg1 antibody was divided into two equal aliquots. One of the aliquots was incubated to a PVDF membrane containing the immobilized *C. albicans* purified Nrg1 protein overnight at 4 °C on a roller. To verify if the *C. albicans* Nrg1p depleted antibody can still detect *C. auris* Nrg1p, one set of cytosolic proteins from *C. auris* (clades I & II) was analyzed by Western blot (left panel). The second set of the identical blot was analyzed in parallel with the original (undepleted) anti-Nrg1 antibody (right panel). *C. albicans nrg1* null mutant was also included as a negative control. While the undepleted anti-Nrg1 antibody reacted to a single protein band of about 52 kDa, the depleted antibody failed to show a strong reaction to *C. auris* cellular proteins suggesting the anti-*Cal*Nrg1 antibody recognizes Nrg1p specifically. The anti-GAPDH HRP-conjugate (human) antibody was used as a loading control.

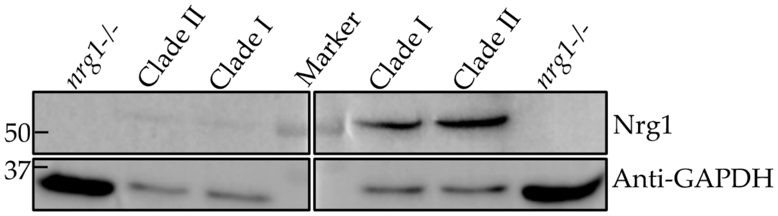

Nrg1p depleted antibody

Nrg1 antibody
